# Application of the ESMACS Binding Free Energy Protocol to a Multi‐Binding Site Lactate Dehydogenase A Ligand Dataset

**DOI:** 10.1002/adts.201900194

**Published:** 2019-11-18

**Authors:** David W. Wright, Fouad Husseini, Shunzhou Wan, Christophe Meyer, Herman van Vlijmen, Gary Tresadern, Peter V. Coveney

**Affiliations:** ^1^ Centre for Computational Science Department of Chemistry University College London London WC1H 0AJ UK; ^2^ Computational Science Laboratory Institute for Informatics Faculty of Science University of Amsterdam Amsterdam 1098XH The Netherlands; ^3^ Janssen Research & Development Turnhoutseweg 30 B‐2340 Beerse Belgium

**Keywords:** binding free energy calculations, fragment‐based drug design, molecular dynamics, molecular mechanics Poisson–Boltzmann surface area (MMPBSA)

## Abstract

Over the past two decades, the use of fragment‐based lead generation has become a common, mature approach to identify tractable starting points in chemical space for the drug discovery process. This approach naturally involves the study of the binding properties of highly heterogeneous ligands. Such datasets challenge computational techniques to provide comparable binding free energy estimates from different binding modes. The performance of a range of statistically robust ensemble‐based binding free energy calculation protocols, called ESMACS (enhanced sampling of molecular dynamics with approximation of continuum solvent), is evaluated. Ligands designed to target two binding pockets in the lactate dehydogenase, a target protein, which vary in size, charge, and binding mode, are studied. When compared to experimental results, excellent statistical rankings are obtained across this highly diverse set of ligands. In addition, three approaches to account for entropic contributions are investigated: 1) normal mode analysis, 2) weighted solvent accessible surface area (WSAS), and 3) variational entropy. Normal mode analysis and WSAS correlate strongly with each other—although the latter is computationally far cheaper—but do not improve rankings. Variational entropy corrects exaggerated discrimination of ligands bound in different pockets but creates three outliers which reduce the quality of the overall ranking.

## Graphical abstract

A challenging application of ensemble‐based binding free energy calculations is reported in which the dataset exhibits substantial chemical diversity in ligands and their binding modes. Excellent correlation is obtained between predictions and experimental results, notwithstanding that the ligands vary in size, charge, and binding pockets targeted. Entropic contributions are evaluated and compared using three different methods, none of which improve the overall ranking.

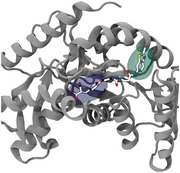

## 1 Introduction

Over the last two decades the use of fragment‐based lead generation (FBLG) has become a common, mature, approach to identify tractable starting points in chemical space for the drug discovery process.^1^ This methodology involves scanning a library of low molecular weight compounds, known as fragments, to see if they bind to the target of interest. Once binding fragments have been identified they can be built on to create higher affinity molecules which, if they modulate protein function as required, become candidate drugs. One possible strategy is to link multiple fragments binding to different regions of the protein. FBLG represents an attractive application for in silico binding affinity calculations, but the need to obtain comparable free energy estimates from different binding modes represents a considerable challenge for many computationally efficient techniques.

Here, we take this challenge to evaluate the performance of our range of ensemble simulation based binding free energy calculation protocols, called ESMACS (enhanced sampling of molecular dynamics with approximation of continuum solvent).

These protocols have been shown to produce results which correlate well with experiment (correlation coefficients >0.7) and provide reproducible uncertainties^2^, ^3^, ^4^, ^5^, ^6^, ^7^ in studies of drugs binding to a single site. ESMACS is based on the common computational binding affinity prediction approach known as molecular mechanics Poisson–Boltzmann surface area (MMPBSA).^8^ This is an approximate post‐processing end‐state method, which uses continuum solvent models to reduce the computational cost of obtaining results. The speed and ease of setup (compared to rigorous free energy calculations) makes MMPBSA an attractive candidate for use throughout the drug discovery pipeline. However, results are generally dependent on the system and binding mode, and are perceived to be less accurate than those obtained from more expensive and theoretically exact alchemical approaches (such as free energy perturbation, FEP, and thermodynamic integration, TI)^9^, ^10^ that have been used successfully in our labs for relative prediction of close analogues in drug discovery.^11^, ^12^, ^13^, ^14^ Furthermore, the term MMPBSA as used in the literature permits a wide range of variants which incorporate different sampling strategies (for example, all ligand conformers can be drawn from simulation of the complex or from independent runs) and different solvation and entropy terms. Our previous work has demonstrated that MMPBSA analysis of single simulation trajectories is highly unreliable, with calculations initiated from the same structures varying by up to 12 kcal mol^−1^ for small molecules bound to proteins.^2^, ^5^, ^15^ Results vary even more significantly for flexible ligands binding to major histocompatibility complex (MHC).^7^ However, the average taken over an ensemble of replica simulations is found to provide reproducible results in good agreement with experimental ranking (*r* >= 0.7) for all these systems. These issues are a result of the underlying lack of reproducibility of classical molecular dynamics, for which predictions of macroscopic properties, such as the Gibbs free energy, require ensemble averaging over microscopic states. Newtonian dynamics are intrinsically sensitive to initial conditions, which manifests as different MD simulations producing trajectories that diverge rapidly over time no matter how close their initial conditions are (a consequence of the mixing ergodic properties of any system that will reach an equilibrium state).^16^ Another limitation of single trajectory MMPBSA is that it does not account thoroughly for entropic components of the binding affinity. Here, we investigate both the use of independent simulations to account for ligand and receptor flexibility, and multiple approaches to incorporating entropic contributions to the binding free energy.

One target in which FBLG has been employed successfully is the lactate dehydogenase (LDH) tetramer, which is upregulated in clinical tumors (with high expression linked to poor prognosis).^17^, ^18^, ^19^ Ward et al.^20^ reported the use of X‐ray crystallography alongside surface plasmon resonance (SPR) and nuclear magnetic resonance (NMR) based screening to develop fragment hits for the LDHA subunit into lead compounds. In this work we report a study testing the performance of ESMACS protocols for datasets including multiple binding sites and ligands varying in size, charge, and binding mode.

## 2 Datasets and Computational Protocols

In this section, we describe the ESMACS protocols we employ in detail and the dataset of 22 LDHA ligands used to test their performance.

### 2.1 Experimental Dataset and Starting Structures

**Figure 1:**
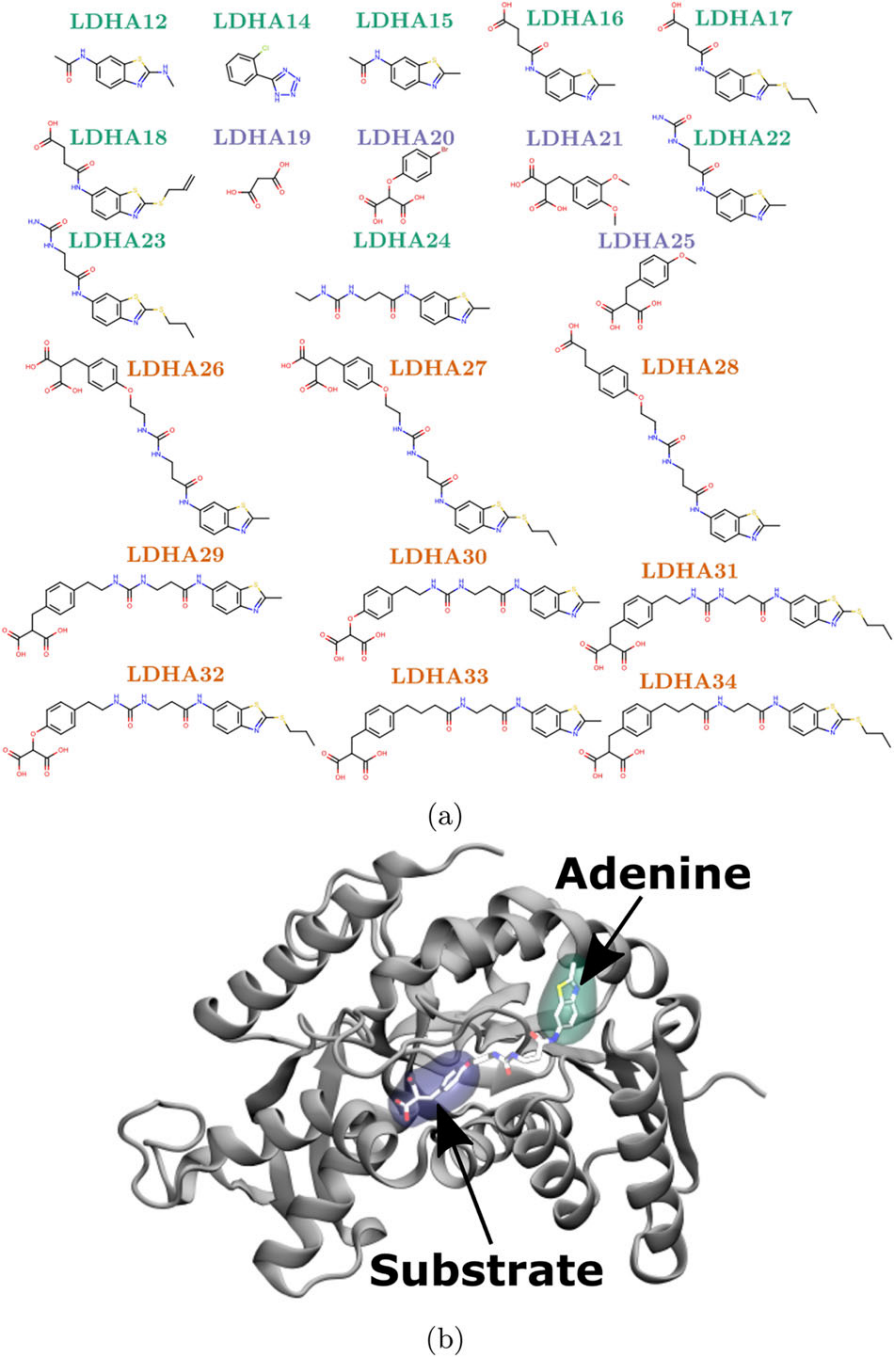
Chemical structures of ligands from the LDHA dataset and locations of binding sites in the protein structure. The ligands bind in one of three modes: to the adenine (green) or substrate (blue) sites individually, or bridging between the two (orange). a) Structures for 22 LDHA ligands. b) A representative bridging ligand (LDHA26) is shown in chemical representation bound to the LDHA protein (shown in cartoon). The moieties bound to the adenine and substrate pockets are highlighted with green and blue surfaces, respectively.

The LDHA structure used in this study is based on chain A of PDB ID: 4AJP (truncated to start at residue 16) with consensus water molecules from other X‐ray structures incorporated. A dataset of 22 ligands was studied, structures of which are shown in **Figure** 1a. The ligands bind to two distinct locations in the protein, known as the substrate and adenine sites, respectively (see Figure 1b). The dataset contains four ligands which bind to the substrate pocket, nine to the adenine pocket, and nine that bridge the two sites. Ligand poses were taken from crystal structures described in Ward et al.^20^ Where no crystal conformation was produced ligands were superimposed on the most similar compound for which an experimental pose was available. This dataset contained both charged and neutral ligands and ligands of growing size. All were neutral except: ligands LDHA14, 16–18, and 28 that were assigned −1 charge, and LDHA19–21, 25–27, 29–34 with a charge of −2. For this data set, binding strengths were obtained from K_*d*_ measurements derived from BIAcore and NMR experiments^20^ and ranged between −11.0 and −3.1 kcal mol^−1^.

### 2.2 ESMACS Binding Free Energy Calculations

The principle behind ESMACS is that many short simulations provide better sampling of conformational space than single simulations. It is based on the MMPBSA method and facilitates rapid and reproducible calculations of binding affinities. Our ESMACS protocols are flexible and allow for the analysis to be tailored to the target system. In this work, we employ a standard ESMACS protocol, which consists of running 25 replicas for a total of 6 ns each, details of which are discussed in Section 2.3.

When two reactants combine at constant temperature and pressure, the binding affinity is characterized by the change in Gibbs free energy, ΔG. This is described by the following relationship:

(1) $$\Delta G=〈G_{\text{complex}}〉-〈G_{\text{receptor}}〉-〈G_{\text{ligand}}〉$$

where $\left\langle G_{\text{complex}}\right\rangle$, $\left\langle G_{\text{receptor}}\right\rangle$ and $\left\langle G_{\text{ligand}}\right\rangle$ are the ensemble average values of the Gibbs free energy for the complex, receptor (protein), and ligand, respectively.

Sampling of the complex and its two components can either be performed independently or derived from simulation of the complex. The latter approach is more commonly used due to its improved convergence behavior, a consequence of cancelation between the noisy terms describing the internal energy of the ligand, receptor and complex.^21^ However, recent work has indicated that adaptation energies associated with confining the receptor and ligand in a complex can differ significantly even for closely related compounds.^12^

When both the receptor and ligand contributions are computed from the complex trajectory, we designate this a “1traj protocol.” When all three contributions derive from independent trajectories we refer to this as a “3traj protocol,” and when only one of the receptor or ligand contributions do so a “2traj protocol.” In the latter case, a suffix (either ‐fl or ‐fr, for flexible ligand and receptor, respectively) is added to the protocol name to signify which component is derived from the independent simulation. Additional variants involve the use of the average receptor contribution across the complex simulations for all comparable ligands, which is indicated with an ‐ar (averaged receptor) suffix in the protocol name.

A summary of all of the protocols and the origin of component data in each is given in **Table** 1. The statistical performance of the pairs of protocols 1traj‐ar and 2traj‐fr, and 2traj‐ar and 3traj, are the same since the receptor contribution for all cases is a constant between protein–ligand pairs. Consequently, we do not analyze the 3traj or 2traj‐fr protocols explicitly.

**Table 1:** Summary of the origin of component contributions in six ESMACS protocols; whether they come from the ensemble of simulations run for the complex (C) or separate ensembles performed for the receptor (R) and ligands (L). “Constant” refers to the use of a constant, usually the average value across the studied systems

	Contribution to the binding free energy		
Protocol	Complex	Receptor	Ligand
1traj	C	C	C
1traj‐ar	C	Constant	C
2traj‐fr	C	R (Constant)	C
2traj‐fl	C	C	L
2traj‐ar	C	Constant	L
3traj	C	R (Constant)	L

The binding free energy change calculated by MMPBSA ($\Delta G_{\text{MMPBSA}}$) can be broken down into a number of components:

(2) $$\begin{array}{c} \Delta G_{\text{MMPBSA}}=\Delta G_{\text{ele}}^{\text{MM}}+\Delta G_{\text{vdW}}^{\text{MM}}+\Delta G_{\text{int}}^{\text{MM}}+\Delta G_{\text{pol}}^{\text{sol}}+\Delta G_{\text{nonpol}}^{\text{sol}} \end{array}$$

where $\Delta G_{\text{ele}}^{\text{MM}}$ and $\Delta G_{\text{vdW}}^{\text{MM}}$ are the electrostatic and van der Waals contributions to the molecular mechanics free energy difference, respectively, $\Delta G_{\text{int}}^{\text{MM}}$ is the internal energy contribution, and $\Delta G_{\text{pol}}^{\text{sol}}$ and $\Delta G_{\text{nonpol}}^{\text{sol}}$ are the polar and non‐polar solvation terms, respectively.

The electrostatic free energy of solvation, $\Delta G_{\text{pol}}^{\text{sol}}$, is the part of the calculation described by the Poisson–Boltzmann (PB) calculation. The *pbsa* program included with AmberTools was used to perform the PB calculation (using default parameters: grid spacing of 0.5 Å, internal and external dielectric constants of 1 and 80, respectively). The non‐polar solvation free energy contribution is estimated from the solvent accessible surface area using the traditional one component method (specified using inp=1 in the input file). In this approach the surface tension, γ, is set to 0.00542 kcal mol^−1^Å^−2^ and the off‐set, β, to 0.92 kcal mol^−1^. The fill ratio parameter was set to 4.0 which does not impact the results but ensures the stability of the calculations. All simulation and analysis in this paper is conducted at a temperature of 300 K.

#### 2.2.1 Entropic Contribution to Binding Free Energies

MMPBSA calculations neglect the contribution to binding free energy from changes in the solute entropy. A computationally expensive method for accounting for this “configurational entropy” is normal mode (NMODE) analysis.^22^ This can straightforwardly be incorporated in the binding affinity estimate using the following equation:

(3) $$\Delta G_{\text{theor}}=\Delta G_{\text{MMPBSA}}-T\Delta S_{\text{NMODE}}$$

The fact that converged normal mode calculations can require similar computational effort to the underlying simulations has motivated the creation of the weighted solvent accessible surface area (WSAS) model.^23^ This model was parameterized to reproduce normal mode results using calculations of the solvent accessible surface area (SAS) and buried SAS (BSAS) of each atom of the system. The two types of surface area are weighted according to atom type, and the sum of the contributions of each atom is used to estimate $S_{\text{WSAS}}$. This estimate is calculated from

(4) $$S_{\text{WSAS}}=\sum_{i=1}^{N}w_{i}\left({\text{SAS}}_{i}-k{\text{BSAS}}_{i}\right)$$

where $w_{i}$ is the atom‐type specific weighting of the atom *i*, and *k* is a parameter which scales the impact of buried surface area. BSAS is calculated from the SAS using

(5) $${\text{BSAS}}_{i}=4\pi {\left(r_{i}+r_{\text{prob}}\right)}^{2}-{\text{SAS}}_{i}$$

Where $r_{i}$ the atomic radius and $r_{\text{prob}}$ the probe radius used to determine the SAS. In this work, we compute SAS using the Lee and Richards algorithm^24^ as implemented in the FreeSASA library (freesasa.github.io).

Another, computationally efficient, alternative approach to accounting for the solute entropy was proposed by Duan et al.^25^ In their formulation, the “variational entropy” can be derived from the fluctuations of the receptor‐ligand interaction energy, $E^{\text{inter}}$. This energy can be calculated using components of the MMPBSA calculation

(6) $$E^{\text{inter}}=G_{\text{ele}}^{\text{MM}}+G_{\text{vdW}}^{\text{MM}}$$

The fluctuation in interaction energy is then given by

(7) $$\Delta E^{\text{inter}}=E^{\text{inter}}-〈E^{\text{inter}}〉$$

where angle braces indicate an ensemble average. This is then used to compute the entropic contribution to binding via

(8) $$-T\Delta S_{\text{var}}=k_{B}T\ln〈e^{\beta \Delta E^{\text{inter}}}〉$$

where $k_{B}$ is the Boltzmann constant and $\beta =1/k_{B}T$.

In this work, we compare the effect of the inclusion of different entropy components on the correlation of computed binding free energy values with experiment. In particular, we study those derived from normal mode analysis, WSAS, and the variational entropy.

#### 2.2.2 Statistics and Uncertainties

All statistics presented are based on their standard definitions with the exception of the mean unsigned error (MUE). It is well known that MMPBSA results have a significant offset from experimental values (typically on the order of 15 to 25 kcal mol^−1^) due to a range of factors, in particular, the neglect of entropic contributions.^26^, ^27^ Consequently, we present values corrected for the systematic (mean signed) error.

We compute uncertainties for all metrics through bootstrapping analysis. This method involves resampling with replacement the *N* input data points (for example, the replica averages of $\Delta G_{\text{MMPBSA}}$) to provide a new bootstrap sample also containing *N* data points. This process is repeated many times (in our case 5000 times) and the statistic of interest calculated for each bootstrap population. The standard deviation of these values provides an estimate of the uncertainty associated with an average derived from a given sample; this is what is quoted as the bootstrap error measure of our statistics. For correlation coefficients, samples are drawn from the overall averages for each ligand paired with the relevant experimental value.

### 2.3 Simulation Setup

Simulation system setup, including the creation of a water box and addition of neutralizing ions, was performed using AmberTools 17.^28^, ^29^ Protein parameters were taken from the standard Amber force field for bioorganic systems (ff14SB).^30^ Ligand parameterizations were produced using the general Amber force field (GAFF).^23^ The primary results of this paper were created using ligand partial charges generated using the restrained electrostatic potential (RESP) procedure, also part of the Amber package, from Gaussian 98^31^ geometrically optimized inhibitor representations. The Gaussian calculations were performed at the Hartree–Fock level of theory using the 6‐31G* basis set. Additional results are presented in which AM1‐BCC partial charges were generated using Amber alone.

Ensembles of 25 replica MD simulations were conducted using the package NAMD 2.11^32^ for each system (complex, receptor, or ligand) studied. All simulations were conducted using the protocol incorporated into the workflow tool BAC.^33^ Each system was minimized with all heavy protein atoms restrained at their initial positions (with a restraining force constant of 4 kcal mol^−1^Å^−2^). Initial velocities were then generated independently for each replica from a Maxwell–Boltzmann distribution at 50 K. Each system was virtually heated to 300 K over 60 ps and subsequently maintained at this temperature using the NAMD standard, Langevin dynamics approach (employing a coupling coefficient of 1 ps^−1^). While heating, the restraints applied during minimisation were retained. Once the system reached the correct temperature, the pressure was maintained at 1 bar using a Berendsen barostat (with a pressure coupling constant of 0.1 ps). Subsequent to heating, a series of equilibration runs, totaling 2 ns, were conducted, during which the restraints on heavy atoms were gradually reduced. The restraint reduction occurred in ten 100 ps steps, after each of which the force constant was halved. Finally, 4 ns production simulations were executed with snapshots output for analysis every 100 ps. For all MD simulation steps the long‐range Coulomb interaction was handled using the particle‐mesh Ewald summation method (PME),^34^ with a nonbonded cutoff distance of 12 Å. The SHAKE algorithm^35^ was employed on all atoms covalently bonded to hydrogen atoms, enabling a 2 fs time step for all simulations.

## 3 Results

First, we investigate the use of ensemble‐based multiple trajectory ESMACS protocols in MMPBSA calculations for this system, before turning our attention to the inclusion of the approaches discussed previously to estimate entropic contributions to the binding energy.

### 3.1 Standard ESMACS Analysis

A comparison of the performance of the various ESMACS protocols in reproducing the LDHA experimental dataset rankings is shown in **Table** 2. All protocols show a strong overall correlation between the computed energies and experimental values, which is reflected in high Pearson and Spearman coefficients. However, some caution should be exercised when considering these metrics as the target dataset consists of three well separated clusters (corresponding to the different binding modes). No significant improvement in either coefficient is made through the incorporation of independent trajectories for ligand or receptor into the calculation, suggesting that the ligands may all bind in a “lock and key” fashion, inducing little if any strain in the protein or change in ligand conformation. As a consequence of this, we will focus for the rest of this work on analyzing the 1traj results.

**Table 2:** Performance of different MMPBSA‐based ESMACS protocols in reproducing experimental binding free energies, measured by mean unsigned error (MUE), Pearson's predictivity index (PI), correlation coefficient (*r* ^2^), and Spearman's rank coefficient ($r_{s}$). Bootstrapped errors are provided in brackets where appropriate

Protocol	MUE^a^	PI	*r* ^2^	$r_{s}$
1traj	17.82	0.90	0.81 (0.07)	0.82 (0.11)
1traj‐ar	22.73	0.90	0.83 (0.07)	0.81 (0.09)
2traj‐fl	16.40	0.91	0.79 (0.07)	0.83 (0.11)
2traj‐ar	21.21	0.90	0.81 (0.06)	0.82 (0.09)

a In kcal mol^−1^ and corrected for mean signed error.

**Figure 2:**
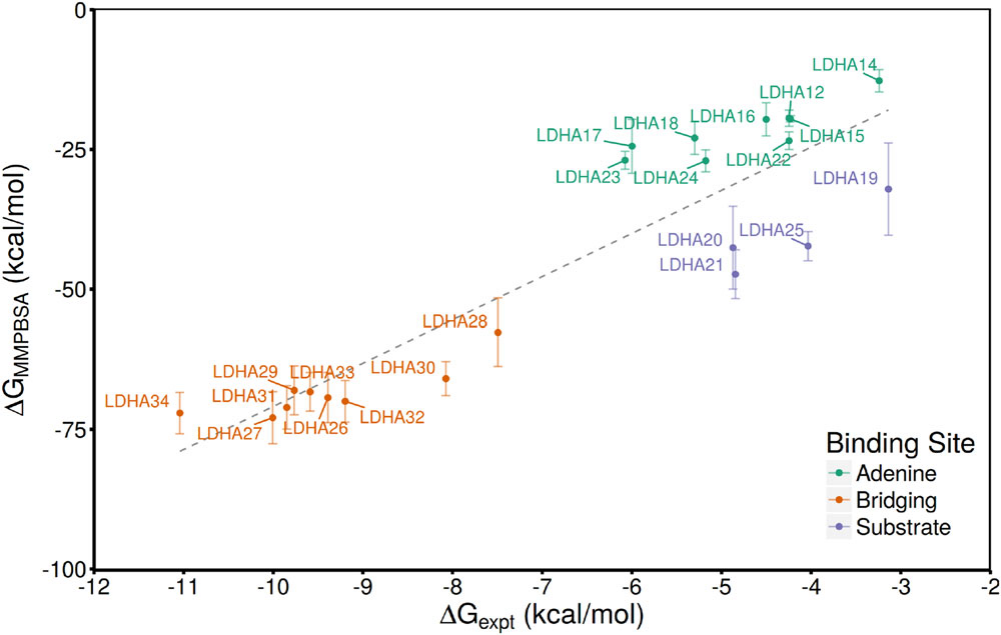
Comparison of binding free energies computed using 1traj MMPBSA based ESMACS protocol with experimental data. Ligand datapoints are colored according to the pocket(s) to which they bind and a dashed gray line indicates the best fit linear regression.

The comparison of binding free energies obtained from the 1traj ESMACS protocols with the LDHA experimental dataset is shown in **Figure** 2. The good quality of the overall ranking is primarily due to correct separation of the tight binding ligands, which occupy both binding sites, and those occupying the adenine site. Consistently good rankings are obtained for each subset of ligands defined by the different binding modes (*r* ^2^ of 0.74 ± 0.17, 0.86 ± 0.24, 0.74 ± 0.16 for the adenine, substrate, and bridging ligands, respectively). This indicates that the method is not merely able to detect the gross changes that differentiate weak (and smaller) binders from tighter (and larger bridging) ligands but accounts for more subtle structural features. The ligands which bind in the substrate pocket (LDHA19, LDHA20, LDHA21, and LDHA25) do not lie close to the overall trend line and are assigned much more favorable relative binding affinities than the adenine site binders, despite having similar experimental affinities. This observation applies to the results of all ESMACS protocols under consideration. These observations are reflected in the high MUE for all rankings shown in Table 2. Graphical comparisons of the results obtained for all protocols with experiment (alongside correlation analysis) are provided in Supporting Information.

Decomposition of the MMPBSA contribution shows that the origin of the improved binding of the ligands that bridge both binding sites compared to the adenine site binders is primarily electrostatic (see **Table** 3). This large negative (attractive) contribution overcomes an increased polar solvation penalty. This is a pattern that is also apparent for the substrate pocket binding ligands. The majority of the increased affinity of the bridging ligands relative to the substrate pocket binders comes from the van der Waals contribution.

**Table 3:** Decomposition of the binding affinity calculated using MMPBSA ($\Delta G^{\text{MMPBSA}}$) into electrostatic ($\Delta G^{\text{ele}}$), van der Waals ($\Delta G^{\text{vdw}}$), polar ($\Delta G^{\text{PB}}$) and non‐polar ($\Delta G^{\text{non-pol}}$) solvation contributions. Ligands which bind in the adenine pocket are shaded green, in the substrate pocket blue and bridging ligands in orange. Values are taken from the 1‐traj ESMACS protocol. Mean energies are in kcal mol^−1^, with bootstrap standard errors shown in parentheses

Drug	$\Delta G^{\text{ele}}$		$\Delta G^{\text{vdw}}$		$\Delta G^{\text{PB}}$		$\Delta G^{\text{non-pol}}$		$\Delta G^{\text{MMPBSA}}$	
LDHA12	−22.24	(2.88)	−26.08	(1.44)	31.70	(2.76)	−2.79	(0.09)	−16.63	(1.37)
LDHA14	−7.38	(3.51)	−20.20	(2.47)	16.95	(2.99)	−2.09	(0.19)	−10.63	(1.91)
LDHA15	−22.07	(1.26)	−24.41	(0.47)	29.64	(1.07)	−2.69	(0.02)	−16.84	(0.55)
LDHA16	−128.05	(20.62)	−27.83	(1.09)	139.37	(18.74)	−3.11	(0.07)	−16.51	(2.93)
LDHA17	−129.44	(23.77)	−32.37	(1.10)	140.88	(21.03)	−3.49	(0.10)	−20.94	(4.80)
LDHA18	−126.25	(17.66)	−31.59	(1.33)	138.33	(16.38)	−3.45	(0.08)	−19.51	(2.89)
LDHA19	−366.15	(16.55)	−4.66	(0.93)	340.18	(12.67)	−1.47	(0.04)	−30.63	(8.27)
LDHA20	−355.68	(18.63)	−21.16	(1.34)	336.91	(15.65)	−2.65	(0.06)	−39.93	(7.46)
LDHA21	−380.30	(14.25)	−24.95	(1.21)	361.13	(12.92)	−3.23	(0.06)	−44.12	(4.39)
LDHA22	−37.48	(2.82)	−31.40	(0.97)	48.87	(2.49)	−3.44	(0.04)	−20.00	(1.57)
LDHA23	−37.42	(4.25)	−35.35	(1.33)	49.65	(3.87)	−3.82	(0.09)	−23.12	(1.59)
LDHA24	−37.90	(1.39)	−38.62	(1.14)	53.48	(2.13)	−3.99	(0.05)	−23.04	(1.95)
LDHA25	−369.56	(18.16)	−22.48	(0.93)	352.67	(16.60)	−2.92	(0.05)	−39.38	(2.63)
LDHA26	−410.61	(17.42)	−56.23	(1.57)	403.40	(14.74)	−5.89	(0.07)	−63.44	(4.51)
LDHA27	−412.59	(17.72)	−59.32	(1.71)	405.24	(15.45)	−6.25	(0.08)	−66.67	(4.63)
LDHA28	−213.51	(10.36)	−55.64	(1.93)	217.22	(6.91)	−5.74	(0.09)	−51.93	(6.13)
LDHA29	−417.16	(12.66)	−53.64	(1.24)	408.53	(9.88)	−5.75	(0.06)	−62.27	(4.38)
LDHA30	−404.65	(14.89)	−54.37	(1.86)	398.85	(13.91)	−5.75	(0.07)	−60.18	(3.08)
LDHA31	−416.20	(12.51)	−57.78	(1.53)	409.05	(10.84)	−6.16	(0.07)	−64.93	(3.91)
LDHA32	−410.65	(12.59)	−57.89	(1.52)	404.70	(11.33)	−6.14	(0.08)	−63.84	(3.74)
LDHA33	−412.50	(10.39)	−54.86	(1.19)	404.86	(10.40)	−5.80	(0.04)	−62.50	(3.47)
LDHA34	−411.27	(10.85)	−59.00	(1.45)	404.40	(9.69)	−6.21	(0.06)	−65.87	(3.70)

#### 3.1.1 Impact of Ligand Parameterization

**Figure 3:**
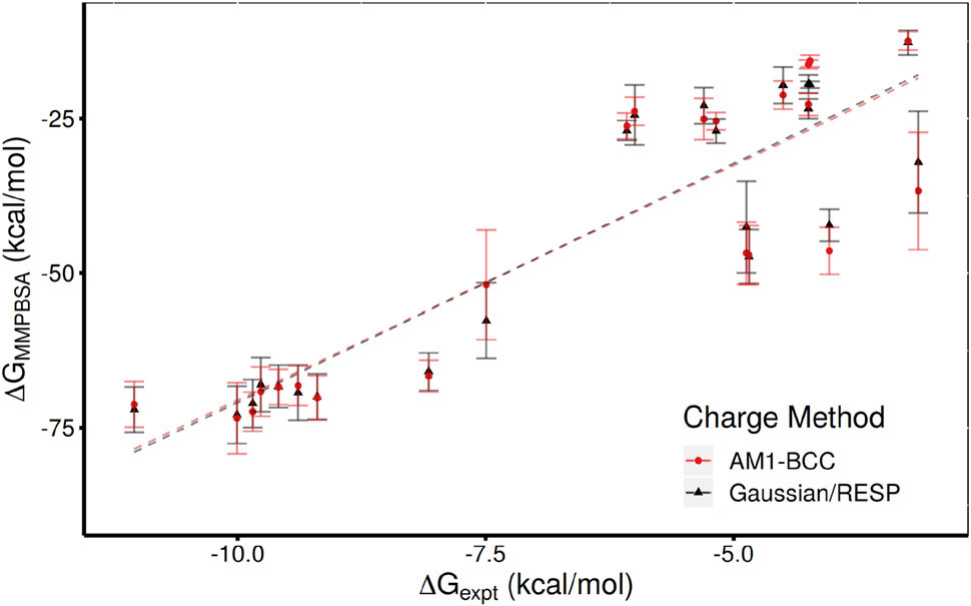
Comparison of binding free energies computed using 1traj MMPBSA based ESMACS protocol and different charge calculation methods with experimental data. Results produced with the AM1‐BCC and Gaussian/RESP approaches agree for all ligands within error except LDHA12 and LDHA15.

For simulations using Amber forcefields, the choice of procedures for ligand preparation is usually whether to use AM1‐BCC or Gaussian/RESP based protocols to determine atom charges in combination with the GAFF general purpose forcefield parameters. In order to ascertain that our results were not strongly influenced by this decision we re‐ran the 1traj ESMACS protocol for the full set of ligands using the AM1‐BCC charge model. As shown in **Figure** 3 the ranking is almost unchanged, with all but two ligands (LDHA12 and LDHA15) having $\Delta G_{\text{MMPBSA}}$ values within error between the two charge models. However, the binding affinities for both ligands have comparatively small uncertainties associated with them. A systematic difference between the partial charges is observed between the two parameterizations in these systems where the oxygen and nitrogen within the binding pockets have the same charges when prepared using Gaussian/RESP (approximately −0.64) but differ slightly (approximately −0.58 and −0.47, respectively) using AM1‐BCC. The correlation coefficients of the results are unchanged, *r* ^2^ is 0.81 (0.07) and $r_{s}$ 0.82 (0.10), with the grouping of the ligands by binding mode also preserved.

### 3.2 Entropic Contributions

Accounting correctly, and computationally efficiently, for the entropic component of binding free energies remains a challenge for MMPBSA‐based computations. Here, we investigate the impact of normal mode analysis derived estimates of the conformational entropy and the variational entropy technique on the ranking of different ESMACS protocols. The latter method is a computationally cheap analysis based on the variation of components of the MMPBSA calculation, while the former involves an expensive extra post‐processing step (obtaining converged values can use as many CPU hours as the original simulation). Consequently, we also investigate the use of the WSAS method for approximating normal mode analysis based on solvent accessible surface area calculations to reduce this added expense.

#### 3.2.1 Normal Mode Analysis and WSAS

**Figure 4:**
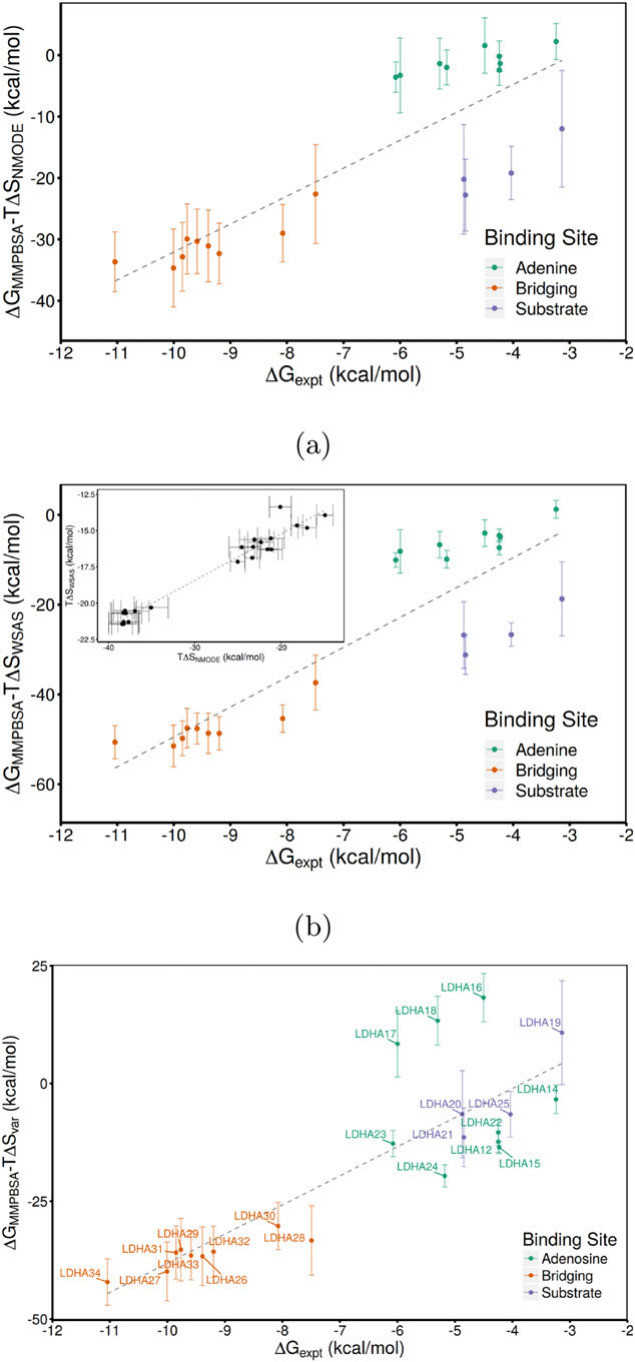
Correlation of ESMACS binding free energies using configurational entropy estimates from a) normal modes analysis, b) WSAS (computed with a k value of 0.8), and c) variational entropy. In all subfigures, the dashed gray line indicates the best fit using linear regression. The correlation between the normal modes and WSAS is shown as an inset in the latter ranking (Pearson correlation (*r* ^2^) of 0.93 and Spearman correlation ($r_{s}$) of 0.94). The ligands bind in one of three modes; to the adenine (green) or substrate (blue) sites individually, or bridging between the two (orange). In the first two methods, the values for substrate and adenine pockets form two distinct clusters, whereas in the variational entropy approach most are in a single region (in agreement with the experimental data).

The overall correlation, shown in (**Figure** 4a), is not improved by the incorporation of normal mode estimates of the configurational entropy (*r* ^2^ of 0.80 and $r_{s}$ of 0.80). Furthermore, the correlations for the ligand binding mode subsets is also similar (see Supporting information). The deviation from the overall trend by the substrate pocket ligands is exaggerated slightly by the incorporation of this contribution with the calculations, particularly for LDHA20 and LDHA21. These ligands are all small and highly charged, building upon a malonate substructure. Interestingly, the ranking of the bridging ligands improves slightly with the incorporation of configurational entropy into the ESMACS calculation, with the weakest binders moving towards the overall trend.

The WSAS method has an exceptionally strong correlation with the normal mode results, with *r* ^2^ of 0.93 and $r_{s}$ of 0.94 (see inset in Figure 4b). Correlation was also good within sets, with *r* ^2^ values of 0.76, 0.83, and 0.98 for adenine, bridging, and substrate subsets, respectively. This is reflected in the similarity of the overall rankings incorporating either method with the MMPBSA results shown in Figure 4. Using the WSAS parameters chosen here, the separation of the bridging ligands to the weakest binding adenine pocket ligands is increased by around 20 kcal mol^−1^. Despite this, the overall pattern of the three binding modes relative to one another is not altered.

These results suggest that, in terms of ranking ligands, the WSAS approach offers a viable “drop in” alternative to normal mode calculations. However, neither approach offers an improvement in this dataset relative to MMPBSA alone. A plausible explanation for the lack of influence of these methods in the LDHA system is the lack of large domain level motions or flexibility.

#### 3.2.2 Variational Entropy

The impact of the addition of variational entropy into the binding free energy is shown in Figure 4c. The main difference made is that the adenine and substrate ligands are brought into line with the exception of ligands LDHA16‐18. This results in a lowered MUE of 13.86, while $r_{s}$ and PI (0.80 and 0.89, respectively) are maintained. However, the outliers reduce *r* ^2^ to 0.71 (0.06).

**Figure 5:**
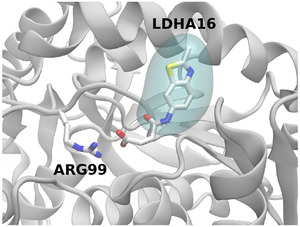
Depiction of interaction of the carboxylic acid moiety of LDHA16 with ARG99 during simulation (both ligand and residue are shown in chemical representation). Ligands LDHA17 and LDHA18 (varying only in substitutions of the methyl sidechain attached to the scaffold rings) share this moiety and these interactions, LDHA12 and LDHA15 have the same scaffold but do not. The region occupied by the shared scaffold, which binds to the main adenine pocket, is shown as a translucent cyan surface. The protein is shown in cartoon representation.

An intuitive explanation for this can be seen in the variability of the $\Delta G_{\text{ele}}^{\text{MM}}$ as described in Table 3, which indicates that the variation in electrostatics is much higher for ligands LDHA16‐18 than for the other adenine pocket binders. The overall electrostatic contribution is also higher owing to the charged carboxylic acid moiety on these ligands. On a structural level, the carboxylic acid protrudes from the main adenine binding site forming transient interactions with ARG99 (see **Figure** 5). Given that the variational entropy is based on the change in interaction energy versus the average, these intuitively lead to changes in the entropic contribution for these molecules. It is likely that in reality these interactions are moderated by solvent effects (including interactions with individual water molecules) not included in the variational entropy calculation. Consequently, it appears that we obtain entropic penalties more in line with those obtained for the more highly charged substrate pocket ligands due to the solvent exposed carboxylic acid. The fact that the binding strength is underestimated indicates that our approach is unlikely to generate false hits but if used uncritically could exclude viable fragments. The larger compounds that span the entire binding site, and the majority of fragments in the substrate or adenine sites, remain well correlated after the inclusion of the variational entropy correction. This is in contrast to our previous work on BRD4^36^ where the same correction was more detrimental for larger compounds, a result we attributed to more movement in their binding site and therefore greater variation in interaction energy during the simulation.

## 4 Conclusions

We obtain excellent statistical rankings across this highly diverse set of ligands which contains considerable variation in size, charge, and binding mode using MMPBSA‐based ESMACS protocols. Such a dataset is not suitable for relative alchemical methods thus ruling out the use of popular FEP or TI methods. Alternatives such as absolute perturbation methods may be plausible, but would also be a challenge given the large and flexible nature of some of the ligands. Therefore, suitable computational methods are needed which motivated us to further test our ESMACS approach, including alternative entropic corrections, and assessing effects of charge models. The fact that the ranking is not improved by the use of multiple trajectories shows that minimal strain was introduced to the protein or ligand upon binding, indicating a “lock and key” binding mechanism for all complexes studied. The research we present here provides a platform for future work which could determine the value of the entropic components investigated here in systems where binding involves greater structural rearrangement (which are best treated using a multiple trajectory approach).

Ligands that bind in different binding sites are separated by MMPBSA binding affinity estimates even when the experimental values are close to one another. This is caused by exaggerated electrostatic interactions in the adenine pocket and bridging ligands compared to those binding the substrate pocket. We investigated whether the separation between groups of ligands could be mitigated via the incorporation of entropic components into the binding free energy estimates. Neither normal mode nor WSAS derived measures of configurational entropy impact the rankings or groupings of ligands, the WSAS ranking correlating strongly with the values obtained from normal mode analysis. However, WSAS can be computed within 30 min on a single core for an entire ensemble whereas normal mode analysis can take up to 12 h to complete and requires one core per snapshot for this duration. Inclusion of variational entropy into the calculations reduces the separation of estimates for drugs binding to the two different binding pockets, but created three outliers which reduced the quality of the overall ranking. The origin of the overestimate of the entropy for these ligands appears to be related to the charge on the solvent exposed carboxylic acid moiety. The difference between the performance of this approach and normal mode analysis or WSAS suggests that relevant states of the LDHA system are distinguished by subtle changes in local interactions rather than global domain motions. In particular, caution must be employed when interpreting results for small ligands in which charged moieties are solvent exposed.

## Conflict of Interest

The authors declare no conflict of interest.

## Supporting information

Supporting Information
