# Supplementary material for: Application of the ESMACS Binding Free Energy Protocol to a Multi‐Binding Site Lactate Dehydogenase A Ligand Dataset
Source: Adv Theory Simul. 2019 Nov 18;3(1):1900194. doi: 10.1002/adts.201900194 (PMC8438761; doi:10.1002/adts.201900194)
Supplement: Supplementary file 1 — Supporting Information [file ADTS-3-1900194-s001.pdf]

Supporting Information: Application of the ESMACS binding free energy protocol to a multi-binding site lactate dehydogenase A ligand dataset

David W. Wright, Fouad Husseini, Shunzhou Wan, Christophe Meyer, Herman van Vlijmen, Gary Tresadern, and Peter V. Coveney

Abstract

In this Supporting Information we provide extra graphical and statistical analysis of the ESMACS protocols applied to the LDHA protein binding dataset studied in the main paper.

Section 1: ESMACS protocol comparison

A variety of MMPBSA[1] based ESMACS[2] protocols were investigated that take into account ligand and receptor flexibility. Figure S1 shows each protocol compared with experiment. No meaningful difference is observable in the clustering of the datapoints across the protocols (including within subsets binding to different sites) - in agreement with the statistical measures described in the main paper.

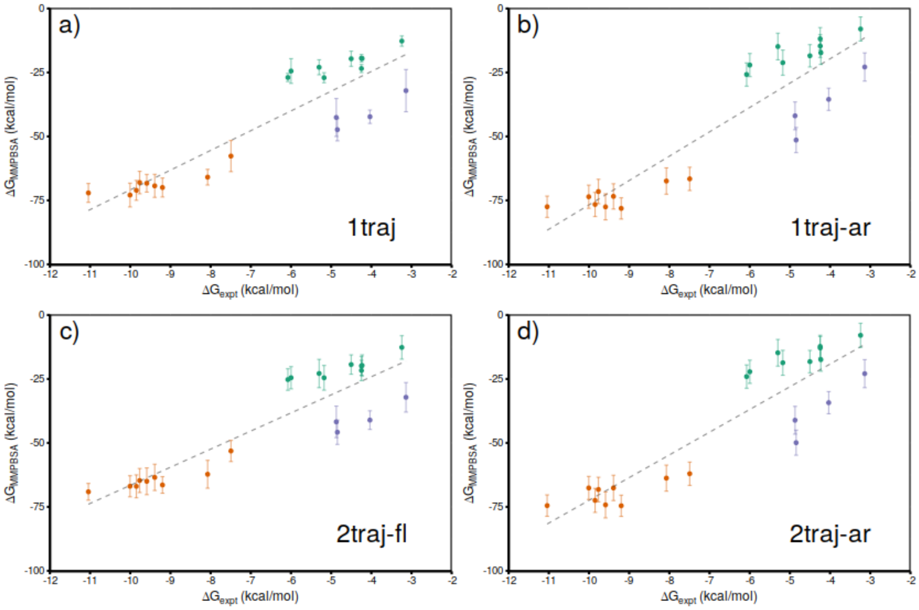

Figure S1: Comparison of binding free energies computed using different MMPBSA based ESMACS protocols with experimental data. Ligand datapoints are coloured according to the pocket(s) to which they bind (adenine pocket in green, substrate pocket blue and bridging ligands orange) and a dashed grey line indicates the best fit linear regression.

Section 2: Statistical analysis of subsets of ligands binding at different sites

The dataset of LDHA ligands under investigation contains 4 ligands which bind to the substrate pocket, 9 to the adenine pocket and 9 that bridge the two sites Ward et al.[3]. In Table S1 the performance of all ESMACS protocols investigated, including those incorporating entropy contributions, are presented both for the whole dataset and the subsets for each binding mode. The slight decrease in performance between the protocols incorporating normal modes and WSAS entropy calculations for the substrate subset is explained by the single outlier visible in the correlation plot shown in the main paper.

Table S1: Performance of different MMPBSA based ESMACS protocols, including 1traj protocols incorporating entropic contributions) in reproducing experimental binding free energies, measured by mean unsigned error (MUE), Pearson's predictivity index (PI), correlation coefficient (r) and Spearman's rank coefficient (r_s). Values are shown for the full dataset and the subsets of ligands binding to different sites. Bootstrapped errors are provided in brackets where appropriate.

**Subset**	**MUE***	**PI**	$\mathbf{r^2}$		$\mathbf{r_s}$	
1traj (MMPBSA alone)						
Overall	17.82	0.90	0.81	(0.07)	0.82	(0.11)
Adenine	2.88	0.84	0.74	(0.17)	0.79	(0.19)
Substrate	3.95	0.99	0.83	(0.24)	0.80	(0.49)
Bridging	2.43	0.86	0.75	(0.17)	0.82	(0.22)
1traj-ar (MMPBSA alone)						
Overall	22.73	0.90	0.82	(0.06)	0.81	(0.09)
Adenine	3.80	0.90	0.76	(0.19)	0.81	(0.22)
Substrate	8.12	0.99	0.89	(0.13)	0.80	(0.50)
Bridging	2.94	0.66	0.60	(0.23)	0.43	(0.39)
2traj-fl (MMPBSA alone)						
Overall	16.40	0.91	0.80	(0.07)	0.83	(0.11)
Adenine	2.25	0.91	0.83	(0.09)	0.85	(0.17)
Substrate	3.48	0.99	0.85	(0.22)	0.80	(0.48)
Bridging	2.36	0.93	0.78	(0.15)	0.87	(0.17)
2traj-ar (MMPBSA alone)						
Overall	21.21	0.90	0.82	(0.06)	0.82	(0.09)
Adenine	3.56	0.90	0.77	(0.19)	0.81	(0.21)
Substrate	7.82	0.99	0.89	(0.12)	0.80	(0.48)
Bridging	3.52	0.74	0.55	(0.24)	0.50	(0.38)
1traj (MMPBSA + Normal modes)						
Overall	11.12	0.89	0.70	(0.11)	0.81	(0.11)
Adenine	1.12	0.85	0.63	(0.22)	0.75	(0.25)
Substrate	2.73	0.99	0.89	(0.18)	0.80	(0.49)
Bridging	1.86	0.86	0.71	(0.19)	0.82	(0.23)
1traj (MMPBSA + WSAS)						
Overall	37.20	0.89	0.82	(0.07)	0.81	(0.11)
Adenine	5.99	0.77	0.65	(0.21)	0.69	(0.25)
Substrate	9.73	0.71	0.72	(0.32)	0.40	(0.69)
Bridging	5.60	0.86	0.73	(0.17)	0.82	(0.22)
1traj (MMPBSA + Variational entropy)						
Overall	13.86	0.89	0.71	(0.09)	0.80	(0.12)
Adenine	11.58	0.03	0.02	(0.16)	-0.11	(0.35)
Substrate	6.56	0.71	0.83	(0.26)	0.40	(0.70)
Bridging	1.77	0.88	0.75	(0.14)	0.78	(0.23)

* In kcal mol^{-1} and corrected for mean signed error.

References

[1] P. A. Kollman, I. Massova, C. Reyes, B. Kuhn, S. Huo, L. Chong, M. Lee, T. Lee, Y. Duan, W. Wang, O. Donini, P. Cieplak, J. Srinivasan, D. A. Case, and T. E. Cheatham. Calculating structures and free energies of complex molecules: combining molecular mechanics and continuum models. *Acc. Chem. Res.*, 33(12):889–897, 2000. doi: 10.1021/ar000033j.

[2] S. Wan, A. P. Bhati, S. J. Zasada, I. Wall, D. Green, P. Bamborough, and P. V. Coveney. Rapid and reliable binding affinity prediction of bromodomain inhibitors: a computational study. *J. Chem. Theory Comput.*, 13(2):784–795, 2017. doi: 10.1021/acs.jctc.6b00794.

[3] R. A. Ward, C. Brassington, A. L. Breeze, A. Caputo, S. Critchlow, G. Davies, L. Goodwin, G. Hassall, R. Greenwood, G. A. Holdgate, M. Mrosek, R. A. Norman, S. Pearson, J. Tart, J. A. Tucker, M. Vodtherr, D. Whittaker, J. Wingfield, J. Winter, and K. Hudson. Design and synthesis of novel lactate dehydrogenase A inhibitors by fragment-based lead generation. *J. Med. Chem.*, 55(7):3285–3306, 2012.
